# Periodontal disease and spontaneous preterm birth: a case control study

**DOI:** 10.1186/1471-2393-6-24

**Published:** 2006-07-19

**Authors:** Stephen Wood, Albert Frydman, Stephen Cox, Rollin Brant, Sheilia Needoba, Barry Eley, Reg Sauve

**Affiliations:** 1 Department of Obstetrics and Gynecology, Foothills Hospital, 1403 29^th ^St. NW., Calgary, Alberta, T2N 2T9, Canada; 2 Suite 403 4600, Crowchild Trail NW, Calgary Alberta, T3A 2L6, Canada; 3 Department of Periodontology, King's College, London Dental Institute at Guy's, King's College and St Thomas' Hospitals, London, SE1 9RT, UK; 4 Center for Community Child Health Research. 4480 Oak Street, L408 Vancouver, BC, V6H 3V4, Canada; 5 46 Waterloo Dr SW. Calgary, Alberta, T3C 3E8, Canada; 6 Department of Community Health Sciences, Health Science Center 3330 Hospital Dr. NW. Calgary, Alberta, T2N 4N1, Canada

## Abstract

**Background:**

Several studies have suggested an association between periodontal disease and prematurity but this finding has not been consistently observed.

**Methods:**

Case control study. Cases (n = 50) were women who had delivered after spontaneous preterm labor at <35 weeks gestation. Two groups of controls (n = 101) were recruited: women who were undelivered but at a preterm gestation and women who delivered at term. A standard, clinical, periodontal examination was performed and gingival crevicular fluid was obtained from standardized locations and tested for neutrophil elastase along with the bacterial enzymes gingipain and dipeptidylpeptidase. Data were analyzed with Fisher's exact tests, ANOVA and multivariate logistic regression.

**Results:**

There was no difference in the proportion of sites with significant attachment loss (≥3 mm): Cases-3.2%, Controls-2.2% p = 0.21. The gingival crevicular fluid concentrations of elastase and gingipain were elevated in cases vs. controls 238.8 uU/*u*l vs. 159.6 uU/*u*l p = .007 and 2.70 uU/*u*l vs. 1.56 uU/*u*l p = .001. On multivariate analysis, the mean log concentration of elastase, but not of gingipain, remained a significant predictor of preterm labor p = .0.015.

**Conclusion:**

We found no evidence that clinical periodontal disease is associated with spontaneous preterm birth. Elevated gingival crevicular fluid levels of elastase were associated with preterm birth but further research is needed before this can be assumed to be a causal relationship.

## Background

Preterm birth remains the most important cause of perinatal mortality and morbidity. Despite considerable research, the pathogenesis of preterm delivery is not well understood and no effective preventative therapy is available. Several, recent studies have suggested a relationship between preterm delivery and periodontal disease. Offenbacher et. al. reported a strong association, OR = 7.9, between periodontal disease and adverse pregnancy outcome defined as either premature delivery or low birth weight in women in North Carolina [1]. Similar results were reported in studies of other U.S. populations by Jeffcoat et. al. and in further work by Offenbacher et al [2,3]. However, this association has not been consistently observed. In fact, Davenport et. al. found that the risk of having a preterm infant was actually reduced in women with periodontitis in the United Kingdom [4].

One concern with these studies is that confounding, especially by socioeconomic status, has not been consistently controlled for. Socioeconomic status has been associated consistently with spontaneous preterm birth and may also be associated with poor dental care, particularly in jurisdictions where it is not publicly funded. As well, no studies of which we are aware, related to spontaneous premature birth, have evaluated biochemical measures of active periodontal disease in addition to standard clinical examination. Since periodontal disease is characterized by a relapsing/remitting pattern, identifying active disease may be an important factor in establishing associations with other disease states such as preterm labor. The population from which the subjects were recruited is also high risk for premature delivery. The city where this study was performed has one of the highest rates of preterm birth in Canada with 10% of births in 2004 occurring before 37 weeks (unpublished local data). The population is predominately Caucasian (81%) with Chinese (6%), South Asian (4%), Aboriginal (2%) and Black (1.5%) comprising the other main ethnic groups [5]. In addition, and in contrast to the studies published to date, our investigation restricted the outcome to only spontaneous preterm birth <35 weeks gestation.

**Figure 1:**
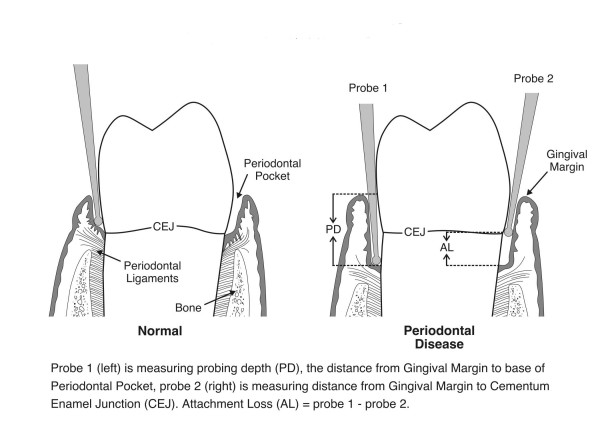
Clinical Examination for Periodontal Disease.

Periodontitis is an inflammatory process initiated by bacterial plaque involving the supporting structures of the tooth which include the gingiva, the junctional epithelium, root cementum, periodontal ligament, and alveolar bone[6]. These structures are responsible for maintaining the attachment of the teeth to the upper and lower jaws. Their destruction leads to loss of attachment between the tooth and the alveolar bone and ultimately, to excess mobility, infection, and loss of the tooth. Periodontitis is diagnosed clinically by measuring a deepening of the space (pocket) between the root of the tooth and the gingival tissue (Figure 1). Attachment loss is an accurate measure of disease severity and is defined as the distance, in mm, between the cemento-enamel junction and the base of the periodontal pocket. As periodontitis is episodic in nature, probing alone cannot determine whether the disease is active or quiescent. Several tests have been developed to assess substances in the gingival crevicular fluid (GCF) which can be obtained from periodontal pockets. One of these tests, which measures neutrophil elastase, has been shown to be highly predictive of eventual attachment loss. Armitage et. al. demonstrated that sites with high neutrophil elastase levels are at significantly greater risk for progressive bone loss over the next 6 months [7]. Using the same elastase substrate in fully quantitative assays, two of the present authors (S.C. and B.E.), in a two year longitudinal study, found that enzyme activity above a critical value had very high sensitivity and specificity for future attachment loss [8]. Further tests based on the two bacterial enzymes dipeptidylpeptidase and gingipain have proved to be of almost equal diagnostic value [9,10].

This case control study was designed to assess the possible relationship between periodontal disease and spontaneous preterm delivery using both clinical examination and the assessment of neutrophil elastase, bacterial gingipain and dipeptidylpeptidase in gingival crevicular fluid.

## Methods

Cases were women with singleton pregnancies who delivered at or before 35 weeks gestation, either after spontaneous labor or induction of labor for preterm premature rupture of membranes. The subjects all had access to universally funded prenatal care but not to publicly funded dental care. Two groups of controls were recruited: a group of postpartum women who delivered at term and the other, undelivered women who were assessed between 22 and 35 weeks gestation. Women were recruited in one hospital following delivery and in the hospital antenatal clinics.

Consenting subjects had a full periodontal examination in a standard, well- equipped, dental office maintained by one of the co-investigators, an experienced periodontist (AF). The dental examination was performed between 2 days and 28 days following delivery for the post partum subjects. Clinical examination was carried out by one highly experienced periodontal hygienist (S.N.). The intra-person variability in clinical examination by the hygienist was assessed by one of the investigators (AF) in a pilot period, using five volunteers, and was found to be acceptably low (mean .34 mm SD .5 mm). Clinical examination included assessment of oral hygiene with a standardized index (Oral Hygiene Index Simplified, OHI-S) [11] and probing for attachment loss. Probing depth and attachment level were measured with a standard probe (UNC-15) for all teeth in 6 locations (disto-lingual, midpoint lingual, mesio-lingual, disto-buccal, midpoint buccal, and mesio-buccal). The number and location of points which bled on probing were noted and a whole mouth bleeding index was calculated as the percentage of all sites. The hygienist was unaware whether the women had delivered prematurely or at term. Samples of gingival crevicular fluid for enzyme analysis were obtained in all subjects except those who had had a course of antibiotics lasting more than one week prior to delivery. This group was excluded because antibiotics could have affected the subgingival microflora and reduced the concentrations of bacterial enzymes. Subjects who had had antibiotics for short courses only during labor were included. The gingival crevicular fluid samples were taken before clinical assessments with 12 × 1.5 mm Whatman chromatography paper #1 with markings every 2 mm. The areas sampled were the typically high risk areas for periodontitis, the mesio-buccal gingival crevices of the two molars and premolars in each quadrant (total of 16 per subject). The supragingival plaque was removed and the sites isolated with cotton wool rolls and air dried. The strips were inserted gently and left in place for 30 seconds. The volume of gingival crevicular fluid was assessed by visually comparing the degree of strip wetting with that produced by known amounts of serum. Although less accurate than electronic measurement [8,9], for the very low volumes of fluid expected from non-inflamed gingivae, visual assessment was considered adequate for the present investigation. This is because local site data were combined for subject mean values in the case-control statistical analysis and the great majority of women (117 of 129 sampled) had fluid volumes >0.1 μl at more than half the sites. Each strip was labeled and placed into 300 *u*L of 50 mM 2-(N-morpholino)-ethane-sulphonic acid (MES) buffer, pH 5.5, with 0.1 mM dithiothreitol (DTT), 0.15 M NaCl and 0.1% v/v Triton X-100. After 1 hour, with occasional agitation at 4°C, the strips were removed and the eluates were frozen with dry ice. The samples were maintained at -20–70°C and transported for analysis in the laboratory of two of the co-investigators (B.E., S.C.) Enzyme activities were determined using selective peptide substrates for neutrophil elastase (MeOSuc-Ala-Ala-Pro-Val-AFC), bacterial gingipain (Z-Val-Lys-Lys-Arg-AFC) and dipeptidylpeptidase (Ala-Pro-AFC). Assay procedures and conditions were as described previously for each enzyme[8,9] Concentrations of liberated 7-amino-4-trifluormethyl coumarin (AFC) were measured after 5 hours with a Perkin Elmer LS30 fluorimeter and enzyme activities were calculated in terms of μUnits pmoles of substrate hydrolysed per minute. Laboratory personnel and the co-investigators performing these analyses were not aware of the subjects' group.

Standardized questionnaires were used to obtain data relating to medical-dental history and socioeconomic status.

Two control groups were used in this study as a previous investigation of periodontal disease in pregnancy had documented decreases in probing depth at term compared to preterm gestations [12]. Therefore, comparisons between postpartum preterm cases and term controls could lead to the observation of a spurious difference. Analysis was planned to examine the control groups separately and only combine them if no obvious difference in attachment loss was observed between these two groups.

Univariate analysis was planned to evaluate mean attachment loss and the frequency of significant attachment loss, (3 mm), between the cases and controls. Final analysis with logistic regression was planned to include possible confounding factors such as smoking, income, and education. Univariate analysis of the log mean enzyme concentrations was also planned as well as a comparison of the number of subjects who had concentrations over critical enzyme levels as this previously had been shown to predict attachment loss [8,9]: neutrophil elastase >400 *u*U/*u*l, bacterial dipeptidylpeptidase >30 *u*U/*u*l and gingipain >30 *u*U/*u*l. Analysis was performed with Stata version 8. A sample size of 50 cases and at least 50 controls was estimated to have at least 80% power to detect an association between clinical periodontitis and preterm birth of the magnitude of an odds ratio equal to 4. The sample size calculation was also based on an estimate of a 13% prevalence of periodontal disease [13].

The study was approved by our institutional ethics review board.

## Results

During the study, we recruited 151 women, 50 cases, 51 undelivered controls and 50 postpartum term controls. One of the undelivered controls delivered preterm, two weeks after her dental examination, so she was reassigned as a case. The remainder of the undelivered controls all subsequently delivered at term.

The 50 cases delivered after spontaneous preterm labor between 22 and 35 weeks gestation (mean = 30.8 +/-3.7 weeks). The mean gestational age of the undelivered controls on examination was 29.2 +/- 4.2 weeks. The post partum controls delivered at a mean gestational age of 39.4 +/-1.1 weeks. Additional characteristics of the subjects are described in Table 1. The Oral Hygiene Index scores for calculus and debris as well as the bleeding index (% of sites with bleeding on probing) were similar amongst the three groups. (Table 2). There was also no difference between the preterm cases and undelivered or postpartum controls in mean attachment loss: 0.86 mm, 0.89 mm, and 0.87 mm respectively, p = .93. The mean percentage of sites probed with ≥ 3 mm of attachment loss was similar amongst the three groups: 3.2% of preterm cases, 2.5% of undelivered controls and 1.9% of postpartum controls, p = 0.33. (Table 2).

**Table 1:** Characteristics of study population.

	Preterm Cases n = 50	Undelivered Controls n = 51	Postpartum term Controls n = 50	p value
Age (years)	30.6 +/- 5.9	30.0 +/- 5.2	32.1 +/- 4.0	0.2*
Nulliparity	20 (40%)	28 (55%)	24 (48%)	0.3*
Post secondary education (years)	2.3 +/- 2.0	4.1 +/- 2.9	4.3 +/- 2.7	<.001*
Gross family income ≤ $20,000.	9 (18%)	4 (8%)	3 (6%)	0.15§
Smoker during pregnancy	13 (26%)	5 (10%)	3 (6%)	0.01§
Last dental cleaning ≤ 6 months	13 (26%)	21 (41%)	21 (42%)	0.12§

Value expressed as mean +/- SD or number (percentage)

*ANOVA §Fisher's exact.

**Table 2:** Full mouth examination data.

	Preterm Cases n = 50	Undelivered Controls n = 51	Postpartum term Controls n = 50	p value
Probing depth (mm)	2.11 +/- .33	2.17 +/- .26	2.14 +/- .21	.68†
Attachment loss (mm)	.86 +/-.32	.89 +/-.26	.87 +/- .18	.93†
Attachment loss ≥ 3 mm (mean %)	3.2 +/- .06	2.5 +/- .04	1.9 +/- .02	.33§
Extent Severity Score (3,5) *	9 (18%)	10 (20%)	4 (8%)	.22§
Debris Score	3.6 +/- 2.6	3.0 +/- 2.1	2.9 +/- 1.7	.42†
Calculus Score	4.5 +/- 3.6	5.1 +/- 3.7	3.9 +/- 2.8	.19†
Bleeding Index (% sites with bleeding)	24 +/- 15	24 +/- 15	20 +/- 11	.41†

Value expressed as mean +/- SD or number (percentage)

* Extent Severity Score (3,5) indicates subjects with = 3 mm of attachment loss at 5% or more of sites probed. † ANOVA §Fisher's exact.

Initial analysis of the attachment loss of the two control groups revealed no significant differences so, as planned, for the final analysis, they were combined. The percentage of sites with attachment loss ≥ 3 mm was 2.2% in the combined control group compared to 3.2% in the preterm cases p = .21. It was originally intended to dichotomize the subjects with an extent severity score of (3,60), which was used by Offenbacher [1]. This would characterize all subjects as having periodontal disease if they were found to have 3 mm or more attachment loss in at least 60% of the sites probed. However, we had no subjects with this degree of periodontal disease. Therefore, as a much lower incidence of periodontitis was encountered, subjects were dichotomized using an extent severity score of (3,5). This characterized the women as having periodontitis if they showed attachment loss ≥ 3 mm in at least 5% of the sites probed, with the threshold corresponding to a moderate level of disease. This lower threshold was also adopted in a recent study [14]. An extent severity score of (3,5) was not associated with preterm labor in the crude analysis OR = 1.36 (0.54, 3.41). Univariate analysis also identified several other factors that were associated with preterm birth: gross family income, education and smoking during pregnancy. Logistic regression analysis was then performed with a model incorporating variables for age, income, smoking during pregnancy, education, dental cleaning within 6 months and periodontal disease defined by an extent severity score of (3,5) or greater. Controlling for a previous preterm birth was considered since it is a well known risk factor for premature birth. However, only one control subject and 7 cases had a previous preterm birth, so it was neither possible nor meaningful to control for this variable. Again, after adjusted analysis, no association between clinical periodontal disease and premature delivery was demonstrated OR = .56 (0.13, 2.37) p = .43. The analysis was repeated for the two control groups separately and again, there was no association with spontaneous premature labor (data not shown).

**Figure 2:**
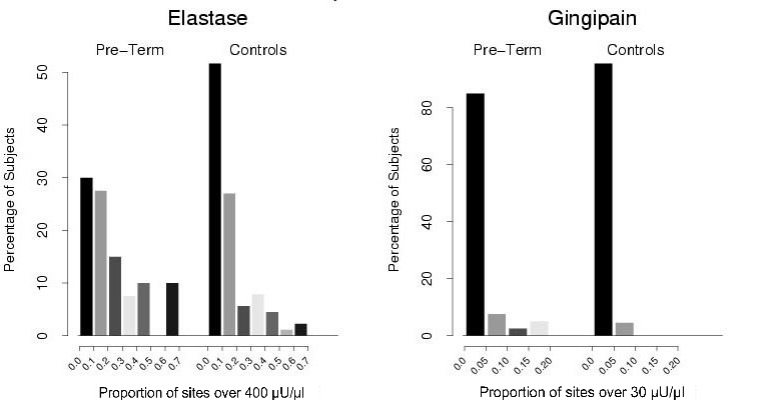
Proportion of subjects by percentage of sites over critical thresholds for gingival crevicular fluid Neutrophil Elastase and Gingipain. Cases vs. combined postpartum and antepartum controls.

Gingival crevicular fluid samples were obtained from sixteen standard sites in 40 of 50 cases, 46 of 51 undelivered controls and 43 of 50 postpartum controls. Mean log concentrations of neutrophil elastase were significantly higher in the cases compared to undelivered and postpartum controls, p = .018 (Table 3). Of the cases, 33 had at least one site with a concentration of neutrophil elastase over the critical value of 400 *u*U/*u*l (range 1–11, median = 3), compared to 30 undelivered controls (range 1–10, median 2) and 32 postpartum controls (range 1–10, median = 2). Mean log concentrations of gingipain were higher in the cases compared to undelivered and postpartum controls, p = .005 (Table 3). Critical gingipain levels (>30 *u*U/*u*l) were observed in 6 preterm cases, three subjects with one high enzyme site, one subject with two and two subjects with three high enzyme sites. Three undelivered controls and one postpartum control subject had one site each with high gingipain levels. There was no significant difference in the mean log concentration of bacterial dipeptidylpeptidase or in the frequency of sites with critical levels between the cases and controls (Table 3). The frequency of gingival crevicular fluid enzyme levels over the critical thresholds for neutrophil elastase and gingipain in cases vs. controls are illustrated in Figure 2.

**Table 3:** Gingival crevicular fluid enzyme levels.

	Preterm Cases n = 40	Undelivered Controls n = 46	Postpartum term Controls n = 43	p value *
**Neutrophil elastase**				
Concentration in GCF. Median (intraquartile range) (uU/*u*l)	261.0 (160.6, 383.1)	180.8 (81.5, 241.6)	169.7 (117.0, 285.4)	0.018
# of subjects with ≥ 1 site with >400 *u*U/*u*l	33	30	32	
**Gingipain**				
Concentration in GCF. Median (intraquartile range) (uU/*u*l)	2.24 (1.38, 3.48)	1.73 (1.01, 2.54)	1.60 (1.0, 2.47)	0.005
# of subjects with ≥ 1 site with >30 *u*U/*u*l	6	3	1	
**Bacterial dipeptidylpeptidase**				
Concentration in GCF. Median (intraquartile range) (uU/*u*l)	1.91 (1.17, 3.40)	2.08 (1.15,2.86)	1.16 (.70, 2.40)	0.192
# of subjects with ≥ 1 site with >30 *u*U/*u*l	5	5	4	

* ANOVA based on differences in mean log concentrations.

Logistic regression analysis was then performed with a model incorporating terms for mean log enzyme levels, age, gross family income, education, smoking during pregnancy and dental cleaning in the last 6 months. In the adjusted analysis, the association between neutrophil elastase concentration and spontaneous premature birth remained significant: OR = 1.93(1.13, 3.28) p = 0.015, but the association with gingipain concentration did not: OR= 3.06 (0.68, 13.8) p = .15. The multivariate analysis was also performed replacing enzyme concentration with a variable for the number of sites with high enzyme levels. The number of high elastase sites was significantly associated with spontaneous preterm birth OR = 1.25 (1.03, 1.52), p = 0.021. The number of high gingipain sites was also positively associated with premature birth but, again, did not reach statistical significance OR = 1.43 (0.36,5.62), p = 0.61. Although the lack of dental cleaning within 6 months of delivery had an association with premature birth in univariate analysis, the association was no longer statistically significant in the final model OR = 1.15 (0.54, 4.26), p = 0.43. Further analysis was then performed to determine if high neutrophil elastase was associated with dental history. It appeared that having a dental cleaning within 6 months and not smoking during pregnancy were highly associated with lower gingival crevicular fluid elastase concentrations (ANOVA, p = .02 and p = .025 respectively). Additional regression analysis was also performed to confirm that the time from delivery to dental examination was not associated with neutrophil elastase levels p = .28.

## Discussion and conclusion

Our study did not find an association between clinical periodontitis, measured by attachment loss, and spontaneous preterm birth. This is in contrast to several of the studies to date [1,2,15,16] but is consistent with the reports of Davenport and Moore et al [17,18]. One reason for these discrepancies may be our strict definition of preterm birth to include only those subjects with a preterm birth after spontaneous labor or induced labor due to preterm premature rupture of membranes. Several previous studies have used definitions of outcome that included intrauterine growth restriction or early second trimester miscarriage or iatrogenic preterm birth rather than spontaneous preterm birth alone [1-3,19]. As there is no indication that these diverse problems have a common pathogenesis, the amalgamation of these outcomes for analysis is methodologically questionable. Differences in disease severity between study population and access to prenatal care may also have lead to different findings amongst studies. However, this lack of consistency raises the possibility that the associations observed in previous studies are non-causal.

Another explanation for a negative finding in any observational study is measurement error. We consider it quite unlikely that this could be responsible for our findings as we used only one, highly trained, blinded examiner to assess all the patients. This consistency in examination should reduce variability in measurement and any tendency to bias the results to a null finding. Finally, limited power could be an explanation for our findings. Although our study was relatively small, the degree of attachment loss was almost identical amongst the three groups. In order to detect a statistically significant difference, of the magnitude we observed, we would have required over 8000 subjects. Our study was also more than adequately powered to detect the strong associations that have been reported in the literature to date, especially if our study had observed a similar frequency of severe periodontal disease as those studies. The fact that we did not find the same frequency of disease does not invalidate our findings and suggests that an association between clinically measured periodontal disease and prematurity may not be evident in all populations. However, we do recognize that, given the relatively small size of our study, inadequate power is always a possible explanation for a null finding.

However, we do report an association between a marker of active periodontitis, gingival crevicular fluid neutrophil elastase, and premature delivery. It is difficult to fully evaluate the significance of this finding, especially as we did not find an association between premature delivery and standard clinical measurements of periodontitis. In our study, the gingival crevicular fluid enzymes were measured after delivery. It may be that following a preterm delivery, women are under increased stress and may neglect their dental hygiene. As neutrophil elastase may rise in gingival crevicular fluid with the development of gingivitis, this may be all that is reflected in our data. However, analysis of the Oral Hygiene Index scores between the case and control groups suggested only a slight, non-significant increase in only the debris scores, but not in the calculus scores, in the preterm group. The possibility of selection bias should also be considered in the evaluation of the results of any case control study. However, none of our patients had symptomatic periodontal disease. Therefore, it seems difficult to conceptualize how patients or study nurses could have perceived factors associated with elevated gingival crevicular fluid enzyme levels and have let this influence the probability of enrolment. One explanation for the inconsistency between the clinical and enzyme data is that high elastase levels reflect active periodontal inflammation and this, rather than past attachment loss, is the more important risk factor. If so, it may be that, in our minimally diseased population, the subjects have not had time to developed clinically apparent disease. Based on our data, such a conclusion is reasonable and would be consistent with a recent publication documenting an increased risk of preterm birth with progression of attachment loss during pregnancy [20]. Furthermore, our data suggest that simple treatments such as dental cleaning and avoidance of smoking during pregnancy are associated with lower gingival crevicular fluid elastase levels. A reduction in gingival crevicular fluid elastase and other enzymes with dental cleaning has been recently reported by Figueredo [21]. Therefore, if a causal relationship between early periodontal disease and premature labor is ultimately proven, this may lead to effective preventative strategies.

It is also possible that previous studies and our own enzyme findings have simply detected a yet to be defined generalized susceptibility to infection in women who deliver preterm. This could place women at increased risk for a variety of infections including periodontal disease and ascending chorioamnionitis leading to premature labor. On the other hand, one possible basis of shared risk, cytokine gene polymorphisms, does not appear to be a common factor in preterm birth and periodontitis [14].

In conclusion, in contrast to other authors, we did not find an association between clinical periodontal disease, measured by attachment loss, and spontaneous premature delivery. We did find an association between gingival crevicular fluid enzyme levels and premature delivery, possibly indicating an association between active periodontal disease and spontaneous premature delivery. However, given the lack of agreement between the analysis of the clinical and enzyme data, we feel it would be premature to conclude that a causal relationship exists. Ultimately, because of the difficulty in proving a temporal relationship between exposure and disease, a case control study can rarely, in and of itself, prove causality. Therefore, at this time a recommendation for screening and treatment of periodontal disease in pregnant women, with the aim of preventing premature birth, cannot be made until further research is completed. Of course, it is still recommended that women who are pregnant or planning to become pregnant continue to maintain optimum periodontal health with professional cleanings and meticulous oral hygiene to prevent periodontal disease. Based on our results, future investigators in this area should also consider measuring markers of active periodontal disease and not rely solely on clinical examination.

## Competing interests

The author(s) declare that they have no competing interests.

## Authors' contributions

All authors contributed to the design of the study. AF, RS and SW conceived the study. SW supervised the recruitment. SN carried out the periodontal examinations under the supervision of AF. SC and BM carried out the GCF enzyme analysis. RB performed the statistical analysis. All authors contributed to and approved the final manuscript.

## Pre-publication history

The pre-publication history for this paper can be accessed here:


